# CT-guided paravertebral injection of doxorubicin for treatment of postherpetic neuralgia: a database-based retrospective stratified study

**DOI:** 10.3389/fneur.2023.1258464

**Published:** 2023-09-12

**Authors:** Fan Lu, JiWei Zhong, Hui Liu, Hong Xiao

**Affiliations:** ^1^Department of Pain Management, West China Hospital, Sichuan University, Chengdu, China; ^2^Department of Anesthesiology, West China Hospital, Sichuan University, Chengdu, China

**Keywords:** postherpetic neuralgia, doxorubicin, pain measurement, quality of life, retrospective study

## Abstract

**Objective:**

This study explored the impact of different doeses of doxorubicin in CT-guided transvertebral foraminal injections for postherpetic neuralgia (PHN) treatment and the impact of 0.5% doxorubicin treatment on patients with different disease courses and lesion locations.

**Methods:**

This retrospective study included 291 patients with PHN who received CT-guided doxorubicin injection at West China Hospital between April 2014 and February 2020.

**Results:**

A total of 228 patients received 0.5% doxorubicin treatment and 63 received 0.33% doxorubicin. Both groups showed significantly improvement in visual analogue scale (VAS) and Brief Pain Inventory (BPI) scores. The 0.5% doxorubicin group demonstrated significant lower VAS scores at 6 and 12 months after surgery (all *p* < 0.001) and a significant lower score on the BPI at 6 and 12 months than the 0.33% doxorubicin group (all *p* < 0.05). Stratified analysis of 0.5% doxorubicin demonstrated a significant reduction in VAS score at 1 week, 3 months, 6 months, and 12 months after treatment compared to baseline (all *p* < 0.05) and significant improvements in BPI score after treatment compared to baseline (*p* < 0.05). The VAS score of the chest group was significant higher than facial, neck and upper limbs and abdomen groupsin groups 1 week after surgery (all *p* < 0.05). Various aspects of quality of life, including daily life, enjoyment of life, sleep, relationships, work, walking ability, and emotions, significantly decreased after surgery (*p* < 0.05). Especially in sleep duration, there was an increase in patients reporting intermediate sleep (4–7 h) and a proportion achieving a normal sleep duration of ≥7 h. And no significant differences of BPI were observed among different affected locations. The incidence of adverse events in the 0.5% doxorubicin group and 0.33% doxorubicin group was 8.78 and 6.34%, respectively.

**Conclusion:**

CT-guided doxorubicin injection therapy has the potential to alleviate pain and enhance the quality of life in patients with PHN. Notably, the use of a 0.5% doxorubicin concentration yields more pronounced pain relief compared to a 0.33% concentration. While longer durations of PHN and specific affected sites may influence the response to treatment, the overall improvements in quality of life remain consistent.

## Introduction

Postherpetic neuralgia (PHN) is a chronic and recurrent neuropathic pain condition that occurs after the healing of herpes zoster (HZ) lesions (1).It affects approximately 5–30% of individuals who have experienced HZ, with 30–50% enduring persistent pain for at least 1 year (2). PHN significantly compromises patients’ quality of life, often resulting in sleep disturbances, mental fatigue, cognitive decline, and depression (3). In severe cases, PHN can even contribute to suicidal tendencies, particularly among the elderly population (4). Hence, it is imperative to explore effective therapies to alleviate the chronic pain associated with PHN. However, the current systemic drug treatments for neuropathic pain, as recommended by guidelines (5–7), have not yielded satisfactory outcomes (8). Despite continuous multi-drug treatments, approximately 66.4% of elderly PHN patients continue to suffer from persistent pain (9).

Doxorubicin is a widely employed chemotherapeutic agent that inhibits DNA replication and induces cell death in cancer treatment (10). It has also shown potential in managing neuropathic pain conditions (11, 12). It is reported that it has been used in conditions like trigeminal neuralgia, intercostal neuralgia, and PHN (11, 13). CT-guided injections, which accurately deliver medication to the affected area, have been used in PHN treatment (11). Nevertheless, the optimal concentration of doxorubicin for CT-guided injections in PHN treatment remains uncertain. Commonly employed concentrations range from 0.25 to 1%; however, higher concentrations pose an increased risk of sensory and motor impairments (11, 14). Long-term efficacy data and large-scale studies are lacking, and further investigation is required to explore the sensitivity of patients with different dermatomes and disease durations to doxorubicin treatment.

Therefore, this study aimed to evaluate the impact of different doses of doxorubicin in CT-guided injections for PHN treatment and the impact of 0.5% doxorubicin treatment on patients with varying disease courses and lesion locations.

## Methods

### Study design and patients

This retrospective study collected data from inpatients with PHN who received CT-guided doxorubicin injection treatment. The data was obtained from the clinical study database management system for PHN at West China Hospital, Sichuan University, spanning the period between April 2014 and February 2020. The study received approval from the Institutional Review Board, and informed consent was waived due to the retrospective nature of the study.

The inclusion criteria were as follows: (1) Age > 18 years; (2) Persistence of pain after the resolution of herpes zoster rash for at least 1 month; (3) Presence of rashes distributed in any region of the body; (4) Baseline pain assessed using the visual analogue scale (VAS) with a score of 4 or higher; (5) Patients who underwent CT-guided injection of doxorubicin into the paravertebral or trigeminal semilunar ganglia. The exclusion criteria were as follows: (1) Patients with PHN resulting from multiple episodes of herpes zoster; (2) Patients who underwent other neuromodulation surgeries, such as radiofrequency modulation of the dorsal root ganglion or absolute alcohol block of the intercostal nerve; (3) Individuals experiencing severe pain due to conditions other than PHN, such as secondary neuralgia caused by tumor invasion of the corresponding nerve segment, primary intercostal neuralgia without a history of herpes zoster, or primary trigeminal neuralgia.

### Procedure

Our department’s pain specialists are responsible for conducting thorough pain assessments, providing analgesic treatments, performing interventional procedures, and ensuring the ongoing health surveillance. In this context, patients with PHN underwent an initial pain evaluation to establish their baseline condition, and subsequently, they were assigned to receive doxorubicin injections following careful preoperative preparation. For spinal PHN, the CT guidance was used to locate three adjacent intervertebral foramina in the corresponding segment. A 21G, 10 cm puncture needle was then inserted, and upon reaching the target, a mixture of diprospan and lidocaine was injected. 1 mL of 0.33% or 0.5% doxorubicin was injected into each segment after drug diffusion confirmation. In trigeminal cases, foramen oval localization using a 21G, 15 cm needle under CT guidance was done, and doxorubicin was administered after observing the patient for 5 min without any adverse reactions. Throughout the follow-up and data recording processes, the nurse specialist assessed specific parameters at regular intervals, including pain levels using the VAS, pain characteristics using the DN4 questionnaire, analgesic usage, and the impact of pain on the patient’s quality of life as measured by BPI.

### Data collection and definition

Patient data related to PHN were retrieved from an existing study database by two physicians uninvolved in surgical procedures or follow-up evaluations. The collected information included gender, age, disease duration, lesion location, as well as preoperative and postoperative pain assessment, sleep quality, quality of life (QOL), occurrence of adverse reactions, and medication usage at 1 week, 3 months, 6 months, and 12 months after treatment. However, for the 0.33% doxorubicin group, due to incomplete information in the database, we were only able to obtain data for postoperative 6 months and 12 months regarding the BPI scores.

Pain assessment included VAS for pain intensity and DN4 questionnaire (15) for pain nature (neuropathic pain ≥4). Pain frequency was categorized as persistent, > 20 episodes/day, 10–20 episodes/day, < 10 episodes/day, or none. Postoperative pain area change was categorized as expansion, unchanged, < 50% reduction, or ≥ 50% reduction compared to pre-treatment. Sleep duration was recorded and categorized as short sleep (≤ 4 h), intermediate sleep (> 4 and < 7 h), or normal sleep (≥ 7 h) (16–19). The QOL assessment utilized the BPI scale (20), which consists of 7 items. Each item was rated on a scale from 0 to 10. The total score on the scale ranges from 0 to 70 points. Analgesics consumption, postoperative adverse reactions, complications, and medication status were documented. In cases where two or more medications were used concurrently, it was considered as combination medication. Patients receiving 0.5% doxorubicin injection were stratified based on disease duration: ≤ 3, 4–6, 7–12, or > 12 months (21–25) and affected site: facial (trigeminal nerve); neck and upper limbs (C2-8); chest (T1-6); abdomen (T7-12); and lower limbs (L1-5; S1-5).

### Statistical analysis

Statistical analysis was performed using SPSS 26.0 software (IBM Corp., Armonk, NY, United States). The continuous variables were presented as mean ± standard deviation (SD) and analyzed using Student’s t-test or paired sample *t*-test if meeting a normal distribution or using rank sum test if skewed distributed. The categorical variables were expressed as number and percentage [*n* (%)] and analyzed using the chi-square test. Paired rank sum tests were used to analyze changes within the same group at different follow-up time points for count data and ranked data. Two-sided value of p of less than 0.05 was considered statistically significant.

## Results

### Baseline data of patients

**Figure 1:**
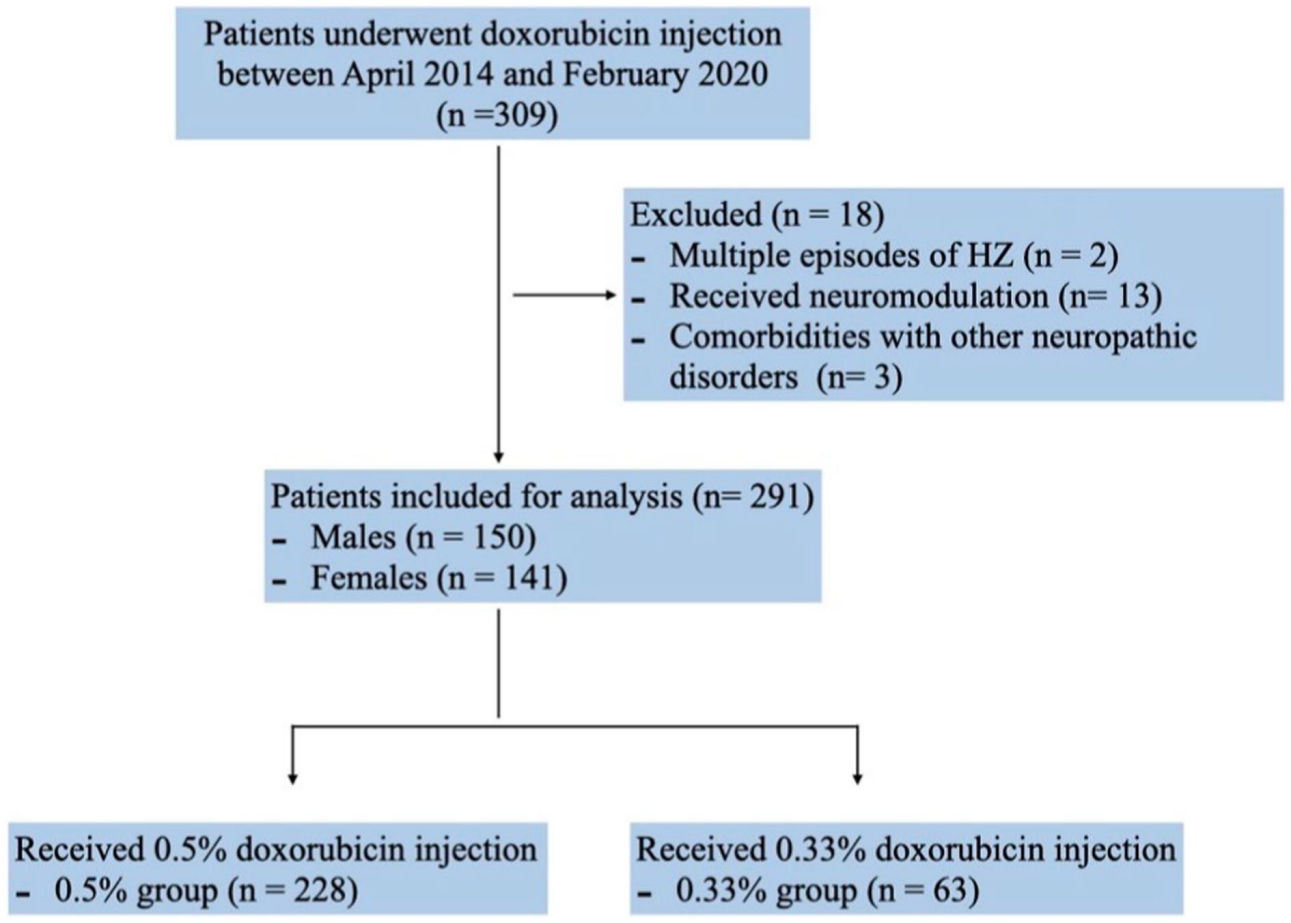
Flowchart of the subject selection.

Between April 2014 and February 2020, a cohort of 309 PHN patients underwent doxorubicin injection with database documentation. Two patients were excluded due to recurrent herpes zoster episodes post-initiation of injection. Additionally, thirteen patients who underwent neuromodulation procedures during follow-up and three with other neuropathic disorders were not included in the analysis. Consequently, the final dataset comprised 291 patients (150 males and 141 females) (Figure 1). Among them, 228 patients received 0.5% doxorubicin treatment while 63 patients received 0.33% doxorubicin treatment. Although the 0.5% doxorubicin group had a slightly longer duration of disease (14.32 vs. 10.4, *p* = 0.048), there was no significant difference in age, gender and the proportion of duration of disease between the 0.5% doxorubicin and 0.33% doxorubicin groups (all *p* > 0.05). The majority of patients in both treatment groups had a disease course of ≤3 months, and the most common disease site was the chest (Table 1).

**Table 1:** Baseline Information.

	Patients treated with 0.5% doxorubicin, *n* = 228	Patients treated with 0.33% doxorubicin, *n* = 63	*p* value
Age (years)	69.14 ± 10.77	68.67 ± 9.61	0.749
Gender, *n* (%)			0.523
Male	117 (51.3%)	33 (52.4%)	
Female	111 (48.7%)	30 (47.6%)	
Duration of disease (months)	14.32 ± 22.87 (0.8 to 120)	10.4 ± 15.37 (1 to 72)	0.048
Duration of Disease, *n* (%)			0.759
≤ 3 months	92 (40.4%)	27 (42.9%)	
4–6 months	31 (13.6%)	10 (15.9%)	
7–12 months	46 (20.2%)	13 (20.6%)	
> 12 months	59 (25.9%)	13 (20.6%)	
Pain dermatome, *n* (%)			0.633
Facial (trigeminal nerve)	19 (8.3%)	0 (0%)	
Neck and upper limbs (C2-8)	44 (19.3%)	18 (28.6%)	
Chest (T1-6)	95 (41.7%)	24 (36.5%)	
Abdomen (T7-12)	67 (29.4%)	17 (27.0%)	
Lower limbs (L1-5; S1-5)	3 (1.3%)	4 (6.3%)	

### Pain assessment

#### Comparison between 0.5 and 0.33% doxorubicin

**Figure 2:**
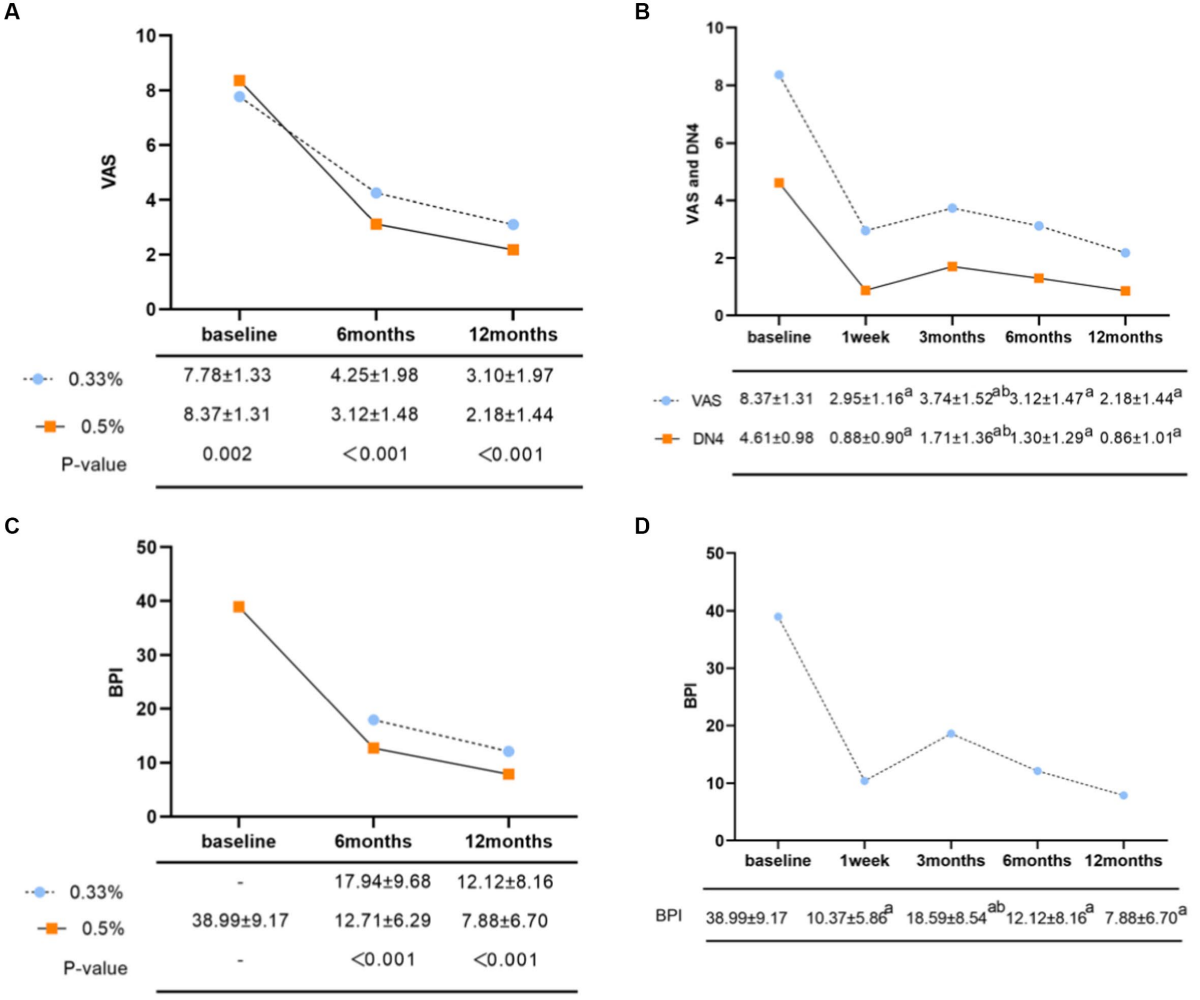
**(A)** Comparison of postoperative VAS scores of different concentrations of doxorubicin. **(B)** Changes in average VAS and DN4 scores in the 0.5% doxorubicin treatment group. ^a^*p* < 0.05, compared with the preoperative group; ^b^*p* < 0.05, compared with the 1-week postoperative group. **(C)** Comparison of total quality of life scores for postoperative BPI with different concentrations of doxorubicin. **(D)** Changes in total BPI scores in the 0.5% doxorubicin group. ^a^*p* < 0.05, compare the postoperative time groups with the preoperative group; ^b^*p* < 0.05, compared with the 3-month postoperative group and the 1-week postoperative group.

The 0.5% group demonstrated significant lower VAS scores than the 0.33% group at 6 and 12 months after surgery (Figure 2A, 3.12 ± 1.48 vs. 4.25 ± 1.98, *p* < 0.001; 2.18 ± 1.44 vs. 3.10 ± 1.97, *p* < 0.001, respectively).

#### Stratified analysis of 0.5% doxorubicin

In terms of pain intensity, the baseline VAS score was 8.37 ± 1.31. At 1 week, 3 months, 6 months, and 12 months after treatment, the VAS scores were 2.95 ± 1.16, 3.74 ± 1.53, 3.12 ± 1.48, and 2.18 ± 1.44, respectively (Figure 2B, *p* < 0.05 compared to the preoperative group). The baseline DN4 score was 4.61 ± 0.98, which decreased to 0.88 ± 0.90, 1.71 ± 1.36, 1.30 ± 1.29, and 0.86 ± 1.01 at 1 week, 3 months, 6 months, and 12 months after surgery, respectively (Figure 2B, *p*< 0.05 compared to the preoperative group). The scores were lowest at 1 week after surgery, gradually increased at 3 months, and then showed a gradual decrease over time. Regarding the pain attacks, approximately 71% of patients experienced persistent or more than 20 episodes pain attacks per day at baseline (Table 2, 36.8% persistent existence; 34.2% >20 times/day). From 1 week to 12 months after surgery, the proportion of patients experiencing pain attacks less than 10 episodes per day increased to 61.8, 64.5, 78.1, and 72.4%, respectively. The proportion of patients with zero pain attack gradually increased from 3 months after surgery, reaching 10.1, 12.7, and 19.7%, respectively (Table 2). In addition, the most common characteristics before surgery were acupuncture sensation (93.0%), tactile-induced pain (87.3%), burning pain (83.8%), and electric shock-like pain (83.8%). We observed that the incidence of postoperative numbness did not significantly decrease at 3 months (24.1%) and 6 months (22.8%) compared to preoperative values (Table 2).

**Table 2:** Pain status of patients in the 0.5% doxorubicin group.

	Baseline	1 week	3 months	6 months	12 months
*Pain frequency*					
Persistent existence	84 (36.8%)	10 (4.4%)	3 (1.3%)	1 (0.43%)	1 (0.43%)
>20 times/day	78 (34.2%)	20 (8.8%)	12 (5.3%)	5 (2.2%)	3 (1.3%)
10–20 times/day	54 (23.7%)	50 (21.9%)	43 (18.9%)	14 (6.1%)	14 (6.1%)
<10 times/day	12 (5.3%)	141 (61.8%)	147 (64.5%)	178 (78.1%)	165 (72.4%)
Zero attack	0 (0%)	7 (3.1%)	23 (10.1%)	29 (12.7%)	45 (19.7%)
*Pain characteristics*					
Burning pain	190 (83.7%)	32 (14.0%)	64 (28.1%)	43 (18.9%)	25 (11.0%)
Cold pain	15 (6.6%)	5 (2.2%)	7 (3.1%)	3 (1.3%)	0 (0%)
Electric shock pain	191 (83.8%)	24 (10.5%)	58 (25.4%)	44 (19.3%)	23 (10.1%)
Tingling	71 (31.1%)	13 (5.7%)	14 (6.61%)	16 (7.0%)	13 (5.7)
Pins and needles	212 (93.0%)	32 (14.0%)	88 (38.6%)	70 (30.7%)	41 (18.0%)
Numbness	66 (28.9%)	39 (17.1%)	55 (24.1%)	52 (22.8%)	35 (15.4%)
Itching	23 (10.1%)	11 (4.8%)	19 (8.3%)	13 (5.7%)	9 (4.0%)
Hypoesthesia to touch	46 (20.2%)	12 (5.2%)	9 (3.9%)	6 (2.6%)	6 (2.6%)
Hypoesthesia to pinprick	38 (16.6%)	7 (3.1%)	4 (1.8%)	6 (2.6%)	6 (2.6%)
Provoked by brushing	199 (87.3%)	22 (9.6%)	76 (33.3%)	43 (18.9%)	22 (9.6%)
*Pain area*					
Expansion	/	3 (1.3%)	0 (0%)	0 (0%)	0 (0%)
Invariant	/	38 (16.7%)	23 (10.1%)	8 (3.5%)	1 (0.43%)
Reduction <50%	/	134 (58.8%)	143 (62.7%)	78 (34.2%)	45 (19.7%)
Reduction ≥50%	/	56 (24.6%)	62 (27.2%)	142 (62.3%)	182 (79.8%)

Regarding pain area, three patients reported an enlargement of the pain area 1 week after surgery. The proportion of patients with a pain area reduction of 50% or more was highest from 6 months to 1 year after surgery (62.3 and 79.8% respectively) (Table 2). In the dermatome stratification analysis, the VAS score of the chest group was significantly higher than the facial, neck, and upper limbs group at 1w but lower than the neck and upper limbs group at 6 m after surgery (*p* < 0.05) (Table 3). Patients with a duration of ≤3 months had significantly higher baseline VAS scores compared to those with a duration of >12 months (8.58 ± 1.39 vs. 8.07 ± 1.40, p < 0.05). However, at 3 months after 0.5% doxorubicin injection, patients with a duration of ≤3 months showed significantly lower VAS scores compared to those with a duration of >12 months (3.48 ± 1.52 vs. 4.24 ± 1.66, *p* < 0.05). At 12 months, patients with a duration of 4–6 months (2.19 ± 1.38) and 7–12 months (2.30 ± 1.50) also had significantly lower VAS scores compared to those with a duration of >12 months (3.07 ± 2.12, *p* < 0.05) (Table 3).

**Table 3:** Changes in VAS scores for different disease duration and dermatomes in the 0.5% doxorubicin group.

	Baseline	1 week	3 months	6 months	12 months
*Duration of disease*					
≤ 3 months	8.58 ± 1.39^a^	2.89 ± 1.14	3.48 ± 1.52^a^	2.90 ± 1.38^a^	2.21 ± 1.47^a^
4-6 months	8.19 ± 1.33	3.23 ± 1.38	3.61 ± 1.26	2.77 ± 1.38^a^	2.19 ± 1.38^a^
7-12 months	8.11 ± 1.10	2.91 ± 1.46	3.80 ± 1.15	3.11 ± 0.99^a^	2.30 ± 1.50^a^
>12 months	8.07 ± 1.40	2.97 ± 0.95	4.24 ± 1.66	3.71 ± 1.70	3.07 ± 2.12
*Pain dermatome*					
Facial	8.21 ± 1.90	2.47 ± 1.02^b^	2.79 ± 1.32^b^	2.58 ± 1.12	1.84 ± 1.34^b^
Neck and upper limbs	8.32 ± 1.25	2.57 ± 0.66^b^	3.36 ± 1.83^b^	3.80 ± 2.01^b^	2.39 ± 1.73
Chest	8.56 ± 1.14	3.35 ± 1.23	4.14 ± 1.34	2.93 ± 1.27	2.95 ± 2.15
Abdomen	8.06 ± 1.13	2.81 ± 1.17^b^	3.73 ± 1.49	3.30 ± 1.49	2.22 ± 1.36^b^
Lower limbs	7.67 ± 2.08	3.33 ± 0.58	3.67 ± 1.53	2.67 ± 0.58	2.67 ± 0.58

a *p* < 0.05, Compare with disease duration > 12 months group.

b *p* < 0.05, Comparison with the chest group.

### QOL assessment

#### Comparison between 0.5 and 0.33% doxorubicin

The BPI in the 0.5% doxorubicin group was lower compared to the 0.33% doxorubicin group at 6 and 12 months after surgery (12.71 ± 6.29 vs. 7.88 ± 6.70; 7.88 ± 6.70 vs. 12.12 ± 8.16), and this difference was statistically significant (*p* < 0.05) (Figure 2C).

#### Stratified analysis of 0.5% doxorubicin

The baseline BPI was 38.99 ± 9.17. Following 0.5% doxorubicin treatment, the BPI scores at 1 week, 3 months, 6 months, and 12 months were 10.37 ± 5.86, 18.59 ± 8.54, 12.12 ± 8.16, and 7.88 ± 6.70, respectively (Figure 2D). Significant differences were observed between the postoperative and preoperative time points (*p* < 0.05). Specifically, the BPI score at 3 months was significantly higher than at 1 week post-surgery (*p* < 0.05). Various aspects of quality of life, including daily life, enjoyment of life, sleep, relationships with others, normal work, walking ability, and emotions, significantly declined after surgery (Table 4, *p* < 0.05). Regarding sleep duration, there was a significant increase in the proportion of patients with intermediate sleep duration (4–7 h). This proportion remained consistent at 61.0–64.0% during the 3–12 months post-surgery. Additionally, the proportion of patients achieving a normal sleep duration (≥7 h) at each follow-up time point after surgery improved, with values of 39.4, 30.7, 22.4, and 30.7% (Table 4).

**Table 4:** Changes in BPI quality of life indicators and sleep duration in patients in the 0.5% doxorubicin group.

	Baseline	1 week	3 months	6 months	12 months
*BPI Indicator*					
Daily life	5.84 ± 1.36	1.55 ± 1.15	2.79 ± 1.39b	1.97 ± 1.39	1.33 ± 1.26
Emotion	6.57 ± 1.84	1.91 ± 1.05	3.29 ± 1.72b	2.07 ± 1.57	1.51 ± 1.33
Walking ability	3.93 ± 1.91	0.96 ± 1.09	1.71 ± 1.18b	1.09 ± 1.00	0.53 ± 0.79
Normal operation	4.88 ± 1.70	1.14 ± 1.06	2.22 ± 1.49b	1.59 ± 1.40	0.79 ± 1.20
Relationship with others	4.89 ± 1.90	1.33 ± 1.00	2.32 ± 1.43b	1.49 ± 1.25	0.89 ± 1.13
Sleep	5.91 ± 1.79	1.64 ± 1.09	3.09 ± 1.67b	1.87 ± 1.31	1.30 ± 1.15
Joy of Life	6.96 ± 1.87	1.83 ± 1.17	3.17 ± 1.67b	2.05 ± 1.61	1.53 ± 1.26
*Sleep duration*					
Short sleep	196 (86.0%)	35 (15.4%)	19 (8.3%)	31 (13.6%)	18 (7.9%)
Intermediate sleep	32 (14.0%)	103 (45.2%)	139 (61.0%)	146 (64.0%)	140 (61.0%)
Normal sleep	0 (0%)	90 (39.4%)	70 (30.7%)	51 (22.4%)	70 (30.7%)

A stratified analysis based on disease course and affected location was performed to assess the impact of 0.5% doxorubicin treatment on quality of life (Table 5). The total BPI score increased with the progression of the disease. Among patients with a disease course exceeding 12 months, the total BPI score was significantly higher than in other groups at various follow-up time points after surgery (*p* < 0.05).When stratifying by dermatome, the total BPI scores in the neck and upper limb groups were higher than in other groups at 6 and 12 months after surgery, with values of 16.36 ± 13.28 and 11.07 ± 10.63, respectively. However, no statistical difference was observed (Table 5, *p* > 0.05).

**Table 5:** Changes in BPI score for different disease duration and dermatomes in the 0.5% doxorubicin group.

	Baseline	1 week	3 months	6 months	12 months
Course of disease					
≤ 3 months	38.83 ± 9.96	9.47 ± 5.96^a^	16.89 ± 7.3^a^	10.16 ± 4.66^a^	6.74 ± 4.91^a^
4–6 months	34.87 ± 6.54^a^	10.35 ± 5.24	14.32 ± 4.09^a^	8.06 ± 3.64^a^	5.45 ± 4.52^a^
7–12 months	42.02 ± 4.48	9.41 ± 3.63^a^	19.09 ± 3.82	12.67 ± 5.75^a^	8.43 ± 4.14^a^
> 12 months	41.62 ± 9.38	12.5 ± 6.33	23.6 ± 10.45	17.41 ± 11.71	12.31 ± 9.59
Pain dermatome					
Facial	33.95 ± 10.86	10.42 ± 8.17	13.37 ± 4.89	8.32 ± 4.40	3.68 ± 3.61
Neck and upper limbs	41.09 ± 10.29	10.20 ± 7.20	18.07 ± 12.51	16.36 ± 13.28	11.07 ± 10.63
Chest	41.02 ± 8.26	11.39 ± 5.24	20.78 ± 7.76	11.35 ± 6.83	8.11 ± 6.21
Abdomen	38.51 ± 8.4	9.49 ± 4.91	19.09 ± 8.35	13.58 ± 7.56	8.28 ± 5.09
Lower limbs	36.67 ± 11.72	15.00 ± 9.54	17.67 ± 2.08	12.67 ± 6.43	8.67 ± 1.16

Comparison between groups with ^a^*p* < 0.05 and disease course > 12 months.

#### Medication usage of 0.5% doxorubicin

Before surgery, the most commonly used drugs among PHN patients were pregabalin (86.4%), oxycodone aminophen (70.2%) and lidocaine cream (35.1%). After the treatment of 0.5% doxorubicin, there was a significant reduction in the proportion of combined medication compared to the pre-surgery period (p < 0.05). The percentage of patients using combined medication at each follow-up time point after surgery was 55.7, 33.8, 19.3, and 5.7%, respectively (Table 6). There was an increase in the proportion of patients using alprazolam, carbamazepine, oxcarbazepine, and non-steroidal analgesics at 3 months after surgery compared to 1 week after the procedure. Furthermore, at 6 and 12 months after the operation, the usage of pregabalin (25.4 and 14.0%) and oxycodone (17.5 and 9.6%) remained higher than that of other drugs (Table 6).

**Table 6:** Medication usage in the 0.5% doxorubicin group.

	Baseline	1 week	3 months	6 months	12 months
Types of drugs					
Non-steroidal analgesics	20 (8.8%)	8 (3.5%)	11 (4.8%)	0 (0%)	0 (0%)
Pregabalin	197 (86.4%)	104 (45.6%)	79(34.6%)	58 (25.4%)	32 (14.0%)
Gabapentin	35 (15.4%)	49(21.5%)	22 (9.6%)	22 (9.6%)	15 (6.6%)
Carbamazepine	20 (8.8%)	13 (5.7%)	19 (8.3%)	10 (4.4%)	3 (1.3%)
Oxcarbazepine	10 (4.4%)	0 (0%)	4 (1.8%)	0 (0%)	2 (0.9%)
Oxycodone and acetaminophen	160 (70.2%)	62 (27.2%)	43 (18.9%)	40 (17.5%)	22 (9.6%)
Tramadol	10 (4.4%)	7 (3.1%)	4 (1.8%)	15 (6.6%)	6 (2.6%)
Lidocaine cream	80 (35.1%)	10 (4.4%)	0 (0%)	0 (0%)	0 (0%)
Antidepressants	16 (7.0%)	0 (0%)	0 (0%)	0 (0%)	0 (0%)
Alprazolam	73 (32.0%)	13 (5.7%)	23 (10.1%)	10 (4.4%)	6 (2.6%)
Combination drug users	215 (94.3%)	127 (55.7%)^a^	77 (33.8%)^a^	44 (19.3%)^a^	13 (5.7%)^a^

a *p* < 0.05, comparison between postoperative time groups and preoperative groups.

### Adverse events

Among the 228 patients with 0.5% doxorubicin treatment, the total incidence of adverse reactions was 8.78%, including 12 cases of postoperative dizziness and nausea, 4 cases of palpitations, and 4 cases of local pain. Additionally, among the 63 patients with the 0.33% doxorubicin treatment, the total incidence of adverse reactions was 6.34%, including 2 cases of dizziness and 2 cases of pain at the puncture site.

## Discussion

The results showed that both the 0.5% doxorubicin and 0.33% doxorubicin groups exhibited significant improvements in VAS and BPI scores following surgery. Moreover, the 0.5% doxorubicin group had significantly lower VAS scores compared to the 0.33% doxorubicin group at 6 and 12 months post-surgery. Notably, the 0.5% doxorubicin group demonstrated a greater reduction in VAS scores while maintaining similar safety profiles. Within the 0.5% doxorubicin group, the lowest scores were observed 1 week after treatment, followed by a slight increase at 3 months, and subsequent decreases over time. Patients with longer disease durations (≥ 12 months) exhibited higher scores. These findings provide evidence of the effectiveness of 0.5% doxorubicin in pain reduction, underscore the importance of early intervention in pain management, and emphasize the need for individualized treatment approaches based on the anatomical location of pain.

This study demonstrated significant improvements in both pain levels and quality of life following CT-guided doxorubicin injection for PHN. The sensation of pain in PHN may be attributed to abnormal firing in nociceptors and low-threshold afferents. Previous research has indicated substantial cellular, axonal, and myelin loss, accompanied by fibrosis, in the sensory ganglia of individuals with severe PHN. By selectively targeting the affected area, doxorubicin has the potential to disrupt the associated signaling pathway and provide pain relief (26). These findings are consistent with a randomized controlled trial investigating the efficacy of doxorubicin in alleviating pain among PHN patients by dorsal root ganglia ablation. Notably, this treatment approach has shown both safety and efficacy, with no reported adverse reactions or significant complications (27).

It is important to note that while 0.33 and 0.5% concentrations are commonly used in doxorubicin injections for PHN (28), limited research has compared their effects. In our study, we observed that patients in the 0.5% doxorubicin group had a longer disease duration and higher VAS scores before treatment. However, despite these initial differences, the 0.5% doxorubicin group exhibited a greater reduction in VAS and BPI scores compared to the 0.33% group. This suggests that 0.5% doxorubicin may have a more pronounced therapeutic effect in relieving pain and improving PHN symptoms.

While a high concentration of doxorubicin has been associated with potential motor neuron damage (29) and an increased risk of cardiac toxicity (30), our study observed a low and comparable incidence of adverse reactions in the 0.5 and 0.33% doxorubicin groups for treating postherpetic neuralgia (PHN) (8.78% vs. 6.34%), which aligns with previous research (28). Importantly, no cases of impaired motor function or sensory disorders were detected. It is crucial to emphasize that the doxorubicin dosage used in PHN treatment was significantly lower than the reported toxic dose, with a maximum dosage of 500 mg/m^2^ (body surface area) implemented to minimize the risk of cardiac toxicity in clinical practice (31). Furthermore, the utilization of CT-guided visualization puncture ensured accurate drug administration to the target location, reducing the possibility of direct bloodstream entry and further decreasing the risk of cardiac toxicity. Therefore, our findings indicate that 0.5% doxorubicin is associated with similar safety compared to 0.33% doxorubicin in the treatment of PHN.

Patients experiencing PHN for more than 12 months showed reduced treatment effectiveness, indicating the challenges posed by persistent neuralgia in PHN. The pathophysiological changes in PHN involve peripheral axonal injury, degeneration of sensory neurons, and atrophy in the spinal dorsal horn (32). These changes occur during the transition from herpes scabbing to the development of PHN. Central sensitization within the central nervous system also plays a crucial role in neuropathic pain (33). As a result, peripheral administration of doxorubicin has limited efficacy in addressing the complex central pain mechanisms associated with long-term PHN. Additional strategies, such as drug therapies, metabolic regulation, pain education, and psychological and behavioral interventions, are necessary to target central sensitization (34). Early administration of doxorubicin has demonstrated positive therapeutic outcomes in PHN, but comprehensive approaches are required for long-term patients. Further research is needed to explore effective strategies for patients with longer durations of PHN.

The effectiveness of doxorubicin injection therapy in relieving pain was found to be less pronounced in the chest group compared to other affected areas. Similarly, patients with involvement in the neck and upper limbs showed less improvement in their quality of life, although this difference was not statistically significant. These findings can be attributed to the frequent involvement of thoracic nerves (T1-T12) in PHN, which affects up to 50% of cases (35). The higher baseline pain intensity and lower quality of life observed in these patients align with previous studies (28). This may be due to heightened attention and sensitivity in these areas, as well as increased friction and tactile pain resulting from upper body activity and respiratory movements. Further research is needed to investigate whether higher doxorubicin doses or alternative methods like Pulsed Radiofrequency Modulation can safely and effectively treat PHN in the chest and back regions.

The study has several limitations. Firstly, its retrospective design introduces inherent limitations, including potential selection bias and lack of control over confounding variables. Secondly, although the sample size was adequate for a single-center study, the generalizability of the results to a broader population may be limited. Additionally, the study duration and follow-up period were relatively short. Future studies with larger, multicenter cohorts and longer follow-up periods are warranted. Lastly, the assessment of pain and quality of life relied on subjective measures, which can be influenced by individual perception and reporting bias. Including objective outcome measures and employing blinding techniques would enhance the validity of the findings. Despite these limitations, the study provides valuable insights into the effectiveness of CT-guided doxorubicin injection therapy in treating PHN, highlighting the need for further investigation and improvements in study design.

## Conclusion

CT-guided paravertebral doxorubicin injection improves pain relief and quality of life in patients with PHN. The effectiveness of 0.5% doxorubicin is superior to 0.33% while ensuring a similar level of safety. Although patients with longer durations and chest affected sites may experience a lower pain relief response, the overall improvement in quality of life remains consistent across treatment segments.

## Data availability statement

The original contributions presented in the study are included in the article/supplementary material, further inquiries can be directed to the corresponding authors.

## Ethics statement

The studies involving humans were approved by the Ethics Committee of West China Hospital, Sichuan University, Chengdu, China. The studies were conducted in accordance with the local legislation and institutional requirements. Written informed consent for participation was not required from the participants or the participants' legal guardians/next of kin in accordance with the national legislation and institutional requirements.

## Author contributions

FL: Conceptualization, Data curation, Formal analysis, Investigation, Methodology, Project administration, Resources, Software, Supervision, Validation, Visualization, Writing – original draft, Writing – review & editing. JWZ: Data curation, Formal analysis, Investigation, Methodology, Project administration, Software, Validation, Visualization, Writing – original draft. HL: Conceptualization, Data curation, Methodology, Project administration, Resources, Supervision, Validation, Visualization, Writing – review & editing. HX: Conceptualization, Methodology, Project administration, Resources, Supervision, Validation, Visualization, Writing – review & editing.

## Funding

This study was supported by grants from the Sichuan Science and Technology Program (No. 2022YFS0300) and West China Hospital of Sichuan University Clinical Research Incubation Project (No. 2021HXFH049).

## Conflict of interest

The authors declare that the research was conducted in the absence of any commercial or financial relationships that could be construed as a potential conflict of interest.

## Publisher’s note

All claims expressed in this article are solely those of the authors and do not necessarily represent those of their affiliated organizations, or those of the publisher, the editors and the reviewers. Any product that may be evaluated in this article, or claim that may be made by its manufacturer, is not guaranteed or endorsed by the publisher.
